# Different dietary carbohydrate component intakes and long-term outcomes in patients with NAFLD: results of longitudinal analysis from the UK Biobank

**DOI:** 10.1186/s12937-023-00897-y

**Published:** 2023-12-08

**Authors:** Zhening Liu, Hangkai Huang, Jiarong Xie, Linxiao Hou, Chengfu Xu

**Affiliations:** 1 Department of Gastroenterology, the First Affiliated Hospital, Zhejiang University School of Medicine, No. 79 Qingchun Road, Hangzhou, 310003 China; 2 Department of Gastroenterology, Ningbo First Hospital, Ningbo, 315010 China; 3 Zhejiang Provincial Clinical Research Center for Digestive Diseases, Hangzhou, 310003 China

**Keywords:** Non-alcoholic fatty liver disease, Carbohydrate quality, End-stage liver disease, Mortality, Substitution

## Abstract

**Background:**

This study aimed to investigate the association between the intake of different dietary carbohydrate components and the long-term outcomes of non-alcoholic fatty liver disease (NAFLD).

**Methods:**

We used prospective data from 26,729 NAFLD participants from the UK Biobank cohort study. Dietary information was recorded by online 24-hour questionnaires (Oxford WebQ). Consumption of different carbohydrate components was calculated by the UK Nutrient Databank Food Composition Table. Cox proportional hazards models were used to estimate the adjusted hazard ratio (HR) and 95% confidence interval (CI). A substitution model was used to estimate the associations of hypothetical substitution for free sugars.

**Results:**

During a median of 10.5 (IQR: 10.2–11.2) years and a total of 280,135 person-years of follow-up, 310 incident end-stage liver disease (ESLD) and 1750 deaths were recorded. Compared with the lowest quartile, the multi-adjusted HRs (95% CI) of incident ESLD in the highest quartile were 1.65 (1.14–2.39) for free sugars, 0.51 (0.35–0.74) for non-free sugars, and 0.55 (0.36–0.83) for fiber. For overall mortality, the multi-adjusted HRs (95% CI) in the highest quartile were 1.21 (1.04–1.39) for free sugars, 0.79 (0.68–0.92) for non-free sugars, and 0.79 (0.67–0.94) for fiber. Substituting free sugars with equal amounts of non-free sugars, starch or fiber was associated with a lower risk of incident ESLD and overall mortality.

**Conclusions:**

A lower intake of free sugars and a higher intake of fiber are associated with a lower incidence of ESLD and overall mortality in NAFLD patients. These findings support the important role of the quality of dietary carbohydrates in preventing ESLD and overall mortality in NAFLD patients.

**Supplementary Information:**

The online version contains supplementary material available at 10.1186/s12937-023-00897-y.

## Introduction

Non-alcoholic fatty liver disease (NAFLD) is a serious public health crisis that poses a burden on 32.4% of the population worldwide [1]. The prevalence of NAFLD has increased over time, propelled by the global epidemics of obesity and type 2 diabetes [2]. NAFLD can progress from simple steatosis to steatohepatitis, fibrosis, cirrhosis and even hepatocellular carcinoma [3]. Cirrhosis currently causes 1.16 million deaths annually, and NAFLD is projected to be the main driver of the rising burden of cirrhosis by 2040 [4, 5]. The incidence of hepatocellular carcinoma [6], liver transplantation, liver-related mortality and all-cause mortality [7] substantially increased with the progression of NAFLD histological stage. Although some drugs, such as vitamin E and pioglitazone, have been shown to improve NAFLD, their adverse effects cannot be ignored. Therefore, management focused on efforts to modify unhealthy lifestyles and mitigate metabolic derangements was considered the standard treatment for NAFLD [8].

Carbohydrates are principally composed of sugars, starch and fiber (non-starch polysaccharides). Sugars may be further categorized as free sugars (those added to foods by the manufacturer, cook or consumer, as well as those naturally present in honey, syrups, and unsweetened fruit juices) or non-free sugars (all sugars excluded from the definition for free sugars, mostly naturally occurring in fruit, vegetables, and dairy products) [9, 10]. Previous attention was mainly paid to the amount of carbohydrate consumption rather than its composition [11]. Low-carbohydrate diets have gained extensive popularity for their weight loss effects, characterized by the replacement of carbohydrates with a higher intake of protein and fat [12]. This dietary pattern is also widely recommended for the dietary control of NAFLD [13]. However, recent studies have demonstrated the detrimental impact of low-carbohydrate diets on all-cause mortality and cardiovascular and cancer-related mortality, which is contradictory to previous findings [14]. The risk of all-cause mortality was higher for both low (< 40%) and high (> 70%) carbohydrate intake than for moderate intake [12]. A meta-analysis of randomized controlled trials showed no significant differences in weight change and cardiovascular risk factors when overweight patients were assigned to a low-carbohydrate or balanced-carbohydrate weight-loss diet [15]. Therefore, recent studies have suggested that carbohydrate components may be more important than carbohydrate quantity. For example, free sugars have been shown to have adverse metabolic outcomes, whereas fiber has been found to be beneficial for health outcomes [10, 16]. Notably, whether NAFLD patients can benefit from altering carbohydrate composition under the same energy intake is unknown. This investigation may provide more strategies for the management of NAFLD.

In this study, we aimed to investigate the association between different components of carbohydrate (free sugars, non-free sugars, starch, and fiber) intake and the risks of end-stage liver disease (ESLD) and all-cause mortality in NAFLD patients. Furthermore, we performed isocaloric substitution between carbohydrate components to determine whether the long-term outcomes of NAFLD would be altered.

## Methods

### Study population

The UK Biobank is a prospective cohort study of over 500,000 participants aged 40–73 years recruited between 2006 and 2010 in 22 assessment centers throughout the UK. At baseline, participants completed a range of information via questionnaires and interviews and provided blood, urine and saliva samples for future analysis. The UK Biobank received ethics approval from the North West Multicenter Research Ethics Committee (reference no. 16/NW/0274). All participants provided written informed consent at recruitment. This research was conducted using the UK Biobank resource under application number 79302.

NAFLD at baseline is identified by the fatty liver index (FLI), which was first proposed in an Italian population and underwent external validation [17, 18]. FLI has an accuracy of 0.84 in detecting fatty liver, and FLI > 60 (positive likelihood ratio = 4.3) indicates the presence of fatty liver. The algorithm is expressed as follows:

$$FLI=\frac{e^{0.953\times \mathit{\ln}(Triglycerides)+0.139\times BMI+0.718\times \mathit{\ln}\left( Gamma\ glutamyltransferase\right)+0.053\times Waist\ circumference-15.745}}{1+{e}^{0.953\times \mathit{\ln}(Triglycerides)+0.139\times BMI+0.718\times \mathit{\ln}\left( Gamma\ glutamyltransferase\right)+0.053\times Waist\ circumference-15.745}}\times 100$$

### Assessment of exposure

As an enhancement to the baseline touchscreen brief FFQ, the Oxford WebQ, a web-based 24-h recall questionnaire, was added to the assessment centers from April 2009 to September 2010 [19]. Moreover, participants who provided a valid email address were invited via e-mail once every 3–4 months to complete the Oxford WebQ between February 2011 and June 2012 (online cycle 1, February 2011 to April 2011; online cycle 2, June 2011 to September 2011; online cycle 3, October 2011 to December 2011; online cycle 4, April 2012 to June 2012). Participants were asked about up to 206 types of foods and 32 types of drinks consumed during the previous 24 hours. Nutrient intakes in this study were calculated using the UK Nutrient Databank (UKNDB) Food Composition Table (2013) [20]. Fiber intake was estimated using the Englyst method [21]. The primary exposure variables were the percentages of total energy intake derived from the three components of carbohydrate [non-free sugars, free sugars, and starch]. Among them, non-free sugars intake was obtained by subtracting free sugars from total sugar. In addition, dietary fiber was displayed as the intake weight (gram). The definition of each component of carbohydrate is shown in Supplementary Table S1. Participants could in fill out the Oxford WebQ on up to five occasions, and we calculated mean values of intake from the available data. All estimated food nutrient data are displayed on the UK Biobank website (Category 100,117).

### Ascertainment of outcomes

The outcomes in this study were incident ESLD and all-cause mortality. The date and cause of hospital admissions were identified through record linkage to Health Episode Statistics for participants from England and Wales and the Scottish Morbidity Records for participants from Scotland. Incident ESLD was defined as a hospital admission or death with ICD-10 (International Classification of Diseases, 10th revision) codes K74.6, K76.6, K76.7, I85.0, I85.9, I86.4, I98.2, I98.3, R18, Z94.4, and C22.0 (Supplementary Table S2). Another outcome of the current study was all-cause mortality. The date of death was obtained from death certificates held within the National Health Service Information Centre (England and Wales) and the National Health Service Central Register (Scotland). At the time of analysis, the updating dates of linkages to hospital inpatient admission and death registries were 30 September 2021 and 31 October 2021, respectively. Follow-up time in person-years was calculated from the beginning of follow-up (the date completed the last Oxford WebQ) until the date of ESLD diagnosis or death, whichever occurred earlier.

### Covariates

Participants completed several touchscreen computer-based questionnaires and then had a face-to-face interview with a trained researcher to provide information on demographic factors (age, sex, ethnicity, education level, and household income) and lifestyle factors (smoking), see Supplementary Table S3. The Townsend deprivation index is an integrated neighborhood-level measure of unemployment, non-car ownership, non-home ownership, and household overcrowding across the UK and was categorized into quintiles from the sample population, with the least deprived (quintile 1) to the most deprived (quintile 5). Sedentary behavior was defined as sedentary time > 4 hours (sum of self-reported hours spent watching TV and using the computer on a typical day). Hypertension was defined as systolic pressure ≥ 140 mmHg, diastolic pressure ≥ 90 mmHg, use of medications for blood pressure or self-reported or diagnosed by a doctor. Diabetes was defined as blood glucose ≥11.1 mmol/L, glycated hemoglobin (HbA1c) ≥48 mmol/mol, use of insulin or self-reported or diagnosed by a doctor. Alanine aminotransferase, triglycerides, and cholesterol levels were measured on a Beckman Coulter AU5800 chemistry analyzer by the UK Biobank. Total energy intake was estimated using the UK Nutrient Databank (UKNDB) Food Composition Table (described above).

### Statistical analysis

**Fig. 1:**
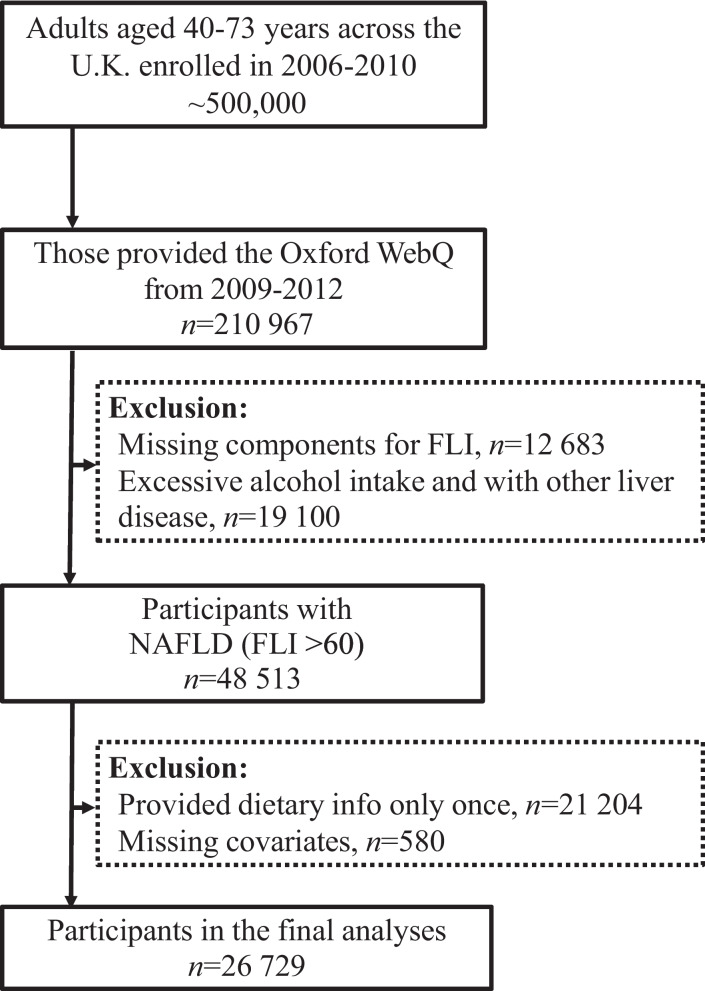
Flow chart of the study design and analytical strategy

There were 210,967 participants who had information on diet. We then excluded patients with excessive alcohol consumption [22] (alcohol consumption ≥ 30 g/d for men and ≥ 20 g/d for women) and those with other liver diseases (viral hepatitis, Wilson’s disease, hemochromatosis, and autoimmune hepatitis); the remaining 48,513 participants (FLI > 60) were diagnosed with NAFLD. Furthermore, due to measurement error and the day-to-day variation, we only included those who completed at least two Oxford WebQ questionnaires (*n* = 26,729, Fig. 1), which had acceptable reproducibility.

Participants were equally divided into four categories according to the consumption of each component of carbohydrates. Categorical variables were displayed as percentages and tested by chi-squared tests. Continuous variables were displayed as the means with standard deviations (SDs) and tested by one-way ANOVA. In the analyses of incident ESLD, we used Fine and Gray competing risk models, with non-liver related mortality as the competing risk. The associations of individual components of carbohydrates with all-cause mortality were investigated using Cox proportional hazard models. Hazard ratios (HRs) and 95% confidence intervals (CIs) for each quartile of exposure were calculated, with the lowest quartile as the reference. Model 1 was adjusted for age, sex, ethnicity, Townsend deprivation index (quintiles), education level (university/college degree or others), household income (less than £18,000, £18,000 to £30,999, £31,000 to £51,999, £52,000 to £100,000, greater than £100,000, or do not know/prefer not to answer), self-reported smoking status (never, former or current smoker), sedentary behavior, body mass index, baseline diabetes, and baseline hypertension. Model 2 was adjusted for model 1 plus serum alanine aminotransferase, triglycerides, cholesterol levels, total carbohydrate intake and total energy intake. Estimates for linear trends were performed by assigning median values to corresponding categories of intake and modeling the values as continuous variables.

In order to estimate the effects of hypothetical substitution of 3% energy of free sugars with the equivalent amounts non-free sugars or starch, we built an isocaloric model by simultaneously including specific component intake (non-free sugars or starch, β_1_) and total intake (non-free sugars plus free sugars or starch plus free sugars, β_2_) in the same model, which also contained other covariates. In this model, the regression coefficient of non-free sugars or starch (β_1_) is interpreted as the theoretical effect of substituting free sugars with equivalent amounts of non-free sugars or starch because their sum (β_2_) is fixed [23]. Since dietary fiber does not provide energy, we explored the equivalent mass of free sugars with incident ESLD and all-cause mortality per substitution of 3 g fiber.

We further investigated whether these associations differed by age, sex, and other factors by performing subgroup analysis. *P* for interaction was tested by introducing a product term of the two variables examined in the regression models. In sensitivity analyses, we further excluded individuals with incident ESLD or who died within 2 years after baseline to avoid reverse causality. Furthermore, to test the robustness of the model, we also excluded those who had extreme BMIs (BMI < 15 or > 40 kg/m^2^), those who reported their previous day’s diet as not being typical and those who had extreme total energy intake (< 800 kcal or > 4500 kcal for men or < 500 kcal or > 3500 kcal for women). SAS 9.4 was used for all analyses. Two-sided *P* values below 0.05 were considered to be statistically significant.

## Results

### Baseline characteristics

A total of 26,729 participants who fulfilled the NAFLD diagnostic criteria were enrolled at baseline. The baseline characteristics of participants by free sugars intake are shown in Table 1. Participants with higher free sugars intake tended to be male, non-white ethnicity, more socially deprived, less educated, and less income. In addition, participants with higher free sugars intake were more often seated, current smokers, had a smaller BMI and waist circumference, and had a lower prevalence of hypertension as well as type 2 diabetes. They also had higher levels of alanine aminotransferase, gamma glutamyltransferase, triglycerides, and total cholesterol. Furthermore, they had more energy intake. The Spearman correlations between the consumption of individual components are presented in Supplementary Table S4.

**Table 1:** Population characteristics stratified by quartiles of free sugars consumption

Variables	Q1 (*n* = 6683)	Q2 (*n* = 6682)	Q3 (*n* = 6683)	Q4 (*n* = 6682)	*P* value
Male (%)	53.9	60.3	65.0	68.6	< 0.001
Age (years)	57.4 ± 7.3	57.3 ± 7.5	57.2 ± 7.6	56.1 ± 8.0	0.001
White ethnicity (%)	96.4	96.2	96.3	95.1	< 0.001
Townsend deprivation index	−1.4 ± 2.9	−1.7 ± 2.8	−1.6 ± 2.9	−1.3 ± 3.0	< 0.001
College or university degree (%)	39.2	40.4	39.4	35.9	< 0.001
Household income (£)					< 0.001
< 18,000	16.8	14.8	16.8	17.7	
18,000 to 30,999	23.1	23.4	23.2	24.1	
31,000 to 51,999	25.9	27.0	26.2	25.8	
52,000 to 100,000	19.8	20.7	20.4	19.2	
> 100,000	5.0	4.6	4.6	4.1	
Sedentary behavior (%)	44.1	41.5	44.2	47.2	< 0.001
Smoking status (%)					< 0.001
Never	52.0	55.2	55.7	55.8	
Previous	41.4	39.5	37.9	35.2	
Current	6.6	5.3	6.4	9.0	
Alcohol consumption (%)					< 0.001
Never or special occasions only	23.6	20.8	20.1	26.0	
1 to 3 times/month	16.7	14.7	15.3	16.7	
1 to 4 times/week	52.4	55.0	54.8	49.1	
Daily or almost daily	7.3	9.5	9.7	8.2	
Body mass index (kg/m^2^)	32.2 ± 4.8	31.4 ± 4.5	31.2 ± 4.4	30.9 ± 4.4	< 0.001
Waist circumference (cm)	103.2 ± 10.4	102.2 ± 9.8	102.0 ± 9.6	102.0 ± 9.7	< 0.001
Hypertension (%)	70.7	68.1	67.8	66.7	< 0.001
Diabetes (%)	17.6	9.9	7.3	5.7	< 0.001
Alanine aminotransferase (U/L)	29.2 ± 16.8	29.1 ± 14.5	29.4 ± 15.7	30.0 ± 16.3	0.007
Gamma glutamyltransferase (U/L)	47.3 ± 44.7	46.6 ± 42.0	47.7 ± 44.4	49.0 ± 44.3	0.014
Triglycerides (mmol/L)	2.3 ± 1.1	2.4 ± 1.1	2.4 ± 1.2	2.5 ± 1.2	< 0.001
Total cholesterol (mmol/L)	5.6 ± 1.2	5.7 ± 1.2	5.7 ± 1.2	5.7 ± 1.2	0.004
Total energy intake (KJ)	8110.8 ± 2095.0	8719.5 ± 2191.7	9004.5 ± 2243.3	9086.1 ± 2377.6	< 0.001

Values are the mean ± standard deviation (SD) or percentage (%) and were examined by one-way ANOVA or chi-square test

Participants excluded from the current analysis were younger, less educated, more socioeconomically deprived, more likely to be women and more likely to have obesity and diabetes, yet their total energy intake was similar (Supplementary Table S5).

### Association of total carbohydrates and different components of carbohydrates with incident ESLD

We first examined the association of total carbohydrate intake and the risk of ESLD and discovered a non-significant association in the multivariable competing risk analysis (Table 2). Then, we explored the association of different components of carbohydrates with incident ESLD and found that free sugars were associated with increased ESLD risk, but non-free sugars and fiber were associated with decreased ESLD risk in NAFLD patients. In the fully adjusted model, for free sugars, compared with the lowest quartile (≤8.2% of energy), the multivariable HRs (95% CI) of incident ESLD in quartile 2–4 were 1.31 (0.93–1.84), 1.26 (0.88–1.81), and 1.65 (1.14–2.39), *P*_trend_ = 0.009. For non-free sugars, compared with the lowest quartile (≤8.8% of energy), the multivariable HRs (95% CI) of incident ESLD in quartile 2–4 were 0.69 (0.50–0.95), 0.70 (0.51–0.97), and 0.51 (0.35–0.74), *P*_trend_ = 0.004. For fiber, the multivariable HRs (95% CI) in quartile 2–4 were 0.83 (0.60–1.13), 0.67 (0.48–0.95), and 0.55 (0.36–0.83), *P*_trend_ = 0.002. However, starch intake showed no significant association with the risk of ESLD.

**Table 2:** HRs of ESLD according to total and different components of carbohydrate

Nutrients	Range^a^ (% energy)	Cases	Model 1		Model 2	
			HR (95% CI)	*P*_trend_	HR (95% CI)	*P*_trend_
Total carbohydrate^b^				0.742		0.852
Q1	≤ 46.3	63	1 (ref)		1 (ref)	
Q2	46.3–50.7	74	1.17 (0.83–1.63)		1.19 (0.85–1.66)	
Q3	50.7–55.1	100	1.13 (0.89–1.42)		1.18 (0.84–1.64)	
Q4	> 55.1	72	1.07 (0.75–1.51)		1.05 (0.74–1.50)	
Free sugars				0.029		0.009
Q1	≤8.2	75	1 (ref)		1 (ref)	
Q2	8.2–11.3	78	1.22 (0.88–1.69)		1.31 (0.93–1.84)	
Q3	11.3–14.9	71	1.16 (0.83–1.63)		1.26 (0.88–1.81)	
Q4	> 14.9	85	1.51 (1.08–2.10)		1.65 (1.14–2.39)	
Non-free sugars				0.007		0.004
Q1	≤8.8	86	1 (ref)		1 (ref)	
Q2	8.8–12.0	70	0.73 (0.53–1.01)		0.69 (0.50–0.95)	
Q3	12.0–15.9	82	0.78 (0.57–1.06)		0.70 (0.51–0.97)	
Q4	> 15.9	71	0.63 (0.45–0.88)		0.51 (0.35–0.74)	
Starch				0.466		0.687
Q1	≤22.2	79	1 (ref)		1 (ref)	
Q2	22.2–25.7	68	0.82 (0.59–1.15)		0.83 (0.59–1.16)	
Q3	25.7–29.3	77	0.95 (0.69–1.31)		0.93 (0.66–1.29)	
Q4	> 29.3	85	0.99 (0.72–1.38)		0.95 (0.66–1.36)	
Fiber				0.001		0.002
Q1	≤13.7	91	1 (ref)		1 (ref)	
Q2	13.7–17.1	87	0.86 (0.64–1.17)		0.83 (0.60–1.13)	
Q3	17.1–21.0	69	0.74 (0.54–1.01)		0.67 (0.48–0.95)	
Q4	> 21.0	62	0.62 (0.45–0.86)		0.55 (0.36–0.83)	

Model 1 was adjusted for age, sex, ethnicity, Townsend deprivation index, education level, household income, self-reported smoking status, sedentary behavior, body mass index, baseline diabetes, and baseline hypertension

Model 2 was adjusted for model 1 plus serum alanine aminotransferase, triglycerides, cholesterol levels, total energy intake and total carbohydrate intake

^a^ Unit for fiber is grams

^b^ Total carbohydrate intake was not adjusted in model 2

### Association of total carbohydrates and different components of carbohydrates with all-cause mortality

Similarly, total carbohydrate intake showed a non-significant association with the risk of all-cause mortality in NAFLD patients (Table 3). Free sugars, non-free sugars, and fiber were all significantly associated with all-cause mortality in NAFLD patients in the fully adjusted model. Compared with the lowest categories, participants in the highest categories had a 21% higher risk of all-cause mortality for free sugars [HR: 1.21 (95% CI, 1.04–1.39), *P*_trend_ = 0.013] and a 21% lower risk of all-cause mortality for non-free sugars [HR: 0.79 (95% CI, 0.68–0.92), *P*_trend_ = 0.002] and fiber [HR: 0.79 (95% CI, 0.67–0.94), *P*_trend_ = 0.003]. Nevertheless, starch intake showed no significant association with the risk of all-cause mortality.

**Table 3:** HRs (95% CIs) of all-cause mortality according to total carbohydrates and different components of carbohydrates

Nutrients	Range^a^ (% energy)	Cases	Model 1		Model 2	
			HR (95% CI)	*P*_trend_	HR (95% CI)	*P*_trend_
Total carbohydrate^b^				0.447		0.458
Q1	≤ 46.3	418	1 (ref)		1 (ref)	
Q2	46.3–50.7	431	1.03 (0.90–1.18)		1.03 (0.90–1.18)	
Q3	50.7–55.1	457	1.09 (0.95–1.24)		1.08 (0.94–1.23)	
Q4	> 55.1	442	1.05 (0.92–1.21)		1.05 (0.92–1.21)	
Free sugars				0.002		0.013
Q1	≤8.2	433	1 (ref)		1 (ref)	
Q2	8.2–11.3	431	1.09 (0.95–1.25)		1.08 (0.95–1.24)	
Q3	11.3–14.9	425	1.10 (0.96–1.27)		1.09 (0.95–1.25)	
Q4	> 14.9	460	1.23 (1.08–1.41)		1.21 (1.04–1.39)	
Non-free sugars				0.015		0.002
Q1	≤8.8	427	1 (ref)		1 (ref)	
Q2	8.8–12.0	454	0.95 (0.83–1.08)		0.93 (0.81–1.06)	
Q3	12.0–15.9	436	0.88 (0.76–1.00)		0.84 (0.73–0.97)	
Q4	> 15.9	431	0.85 (0.74–0.97)		0.79 (0.68–0.92)	
Starch				0.688		0.815
Q1	≤22.2	439	1 (ref)		1 (ref)	
Q2	22.2–25.7	410	0.90 (0.79–1.03)		0.87 (0.76–1.00)	
Q3	25.7–29.3	459	1.02 (0.89–1.16)		0.98 (0.86–1.12)	
Q4	> 29.3	440	0.99 (0.86–1.13)		0.94 (0.82–1.09)	
Fiber				0.279		0.003
Q1	≤13.7	416	1 (ref)		1 (ref)	
Q2	13.7–17.1	443	1.01 (0.89–1.16)		0.95 (0.83–1.09)	
Q3	17.1–21.0	435	0.97 (0.85–1.11)		0.88 (0.76–1.01)	
Q4	> 21.0	434	0.94 (0.82–1.08)		0.79 (0.67–0.94)	

Model 1 was adjusted for age, sex, ethnicity, Townsend deprivation index, education level, household income, self-reported smoking status, sedentary behavior, body mass index, baseline diabetes, and baseline hypertension

Model 2 was adjusted for model 1 plus serum alanine aminotransferase, triglycerides, cholesterol levels, total energy intake and total carbohydrate intake

^a^Unit for fiber is grams

^b^Total carbohydrate intake was not adjusted in model 2

### Substitution analyses

**Fig. 2:**
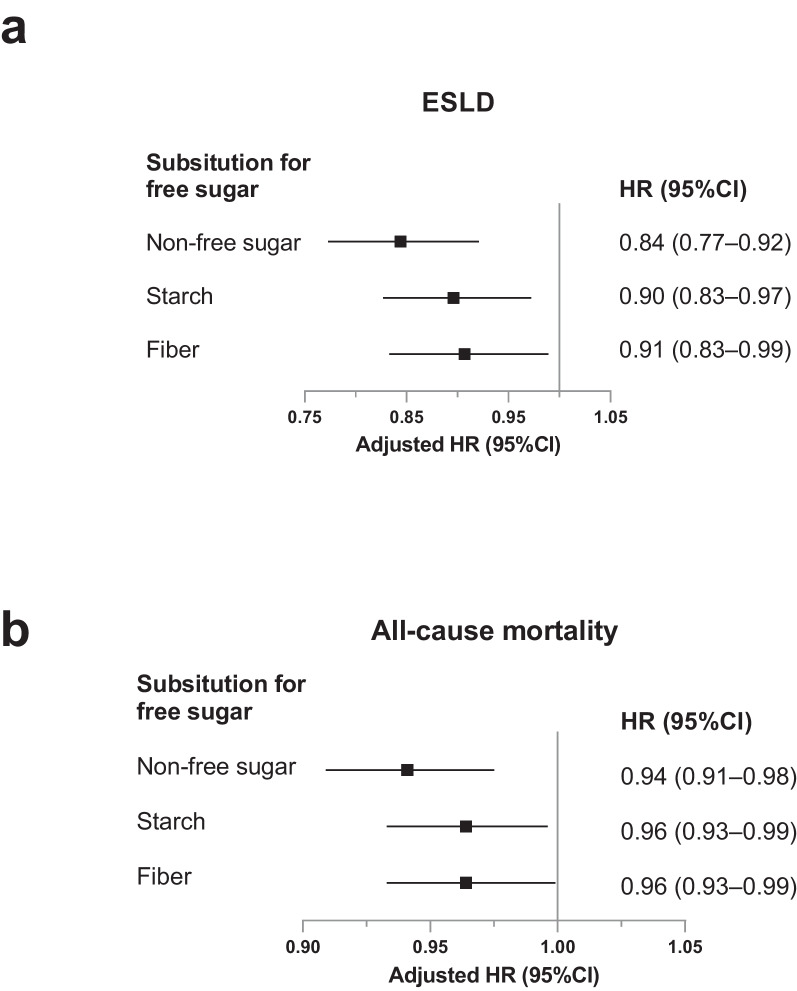
Multivariable-adjusted hazard ratios of incident ESLD and all-cause mortality by substituting free sugars. Forest plots show the multivariable HRs associated with substitution of non-free sugars, starch, and dietary fiber for equivalent amounts of free sugars. HRs were adjusted for age, sex, ethnicity, Townsend deprivation index, education level, household income, self-reported smoking status, sedentary behavior, body mass index, baseline diabetes, baseline hypertension, serum alanine aminotransferase, triglycerides, cholesterol levels, total energy intake, and total carbohydrate intake

Because higher free sugars intake was associated with a higher risk of incident ESLD and all-cause mortality, substitution analysis for this was investigated (Fig. 2). When total sugar intake remained constant, substituting 1 unit (3% energy) of free sugars with equivalent amounts of non-free sugars led to 16 and 6% reductions in the risk of incident ESLD and all-cause mortality, respectively. Similarly, isocaloric replacement (per 3% energy) of free sugars with starch was associated with 10 and 4% reductions in incident ESLD and all-cause mortality, respectively. In addition, we detected a 9% lower risk of incident ESLD and a 4% lower risk of all-cause mortality when replacing 3 g free sugars with equivalent dietary fiber.

### Subgroup analyses and sensitivity analyses

In the subgroup analyses, associations of non-free sugars, free sugars, and fiber consumption with incident ESLD showed no heterogeneity in analyses stratified by sex, age, Townsend deprivation index, education level, smoking status, diabetes, and obesity (Supplementary Tables S6-S8). In addition, the associations of non-free sugars and free sugars with all-cause mortality yielded similar results across subgroups (Supplementary Tables S9 and S10). Notably, a negative association of fiber with all-cause mortality was restricted to those with a Townsend index above the median (*P*_interaction_ = 0.012, Supplementary Table S11).

In sensitivity analyses, the associations of free sugars, non-free sugars, and fiber with incident and all-cause mortality found in the main analyses did not change materially after exclusion of those with incident ESLD or dead in the first 2 years of follow-up or those with extreme BMIs. We detected similar associations when further excluding those who had extreme total energy intake or reported their previous day’s diet as not being typical (Supplementary Tables S12 and S13).

## Discussion

In this prospective cohort study, we explored the association of total dietary carbohydrate intake and its different components with long-term outcomes of NAFLD. We found that total carbohydrate consumption was not significantly associated with ESLD or all-cause mortality risk in patients with NAFLD. Nevertheless, a higher intake of free sugars and a lower intake of non-free sugars or dietary fiber were associated with an increased risk of ESLD and liver-related mortality in NAFLD patients. We also found that isocaloric replacement of free sugars with non-free sugars, starch and fiber was related to reduced risks of adverse outcomes in NAFLD patients.

Several studies have depicted efforts to modify individual carbohydrate consumption to improve hepatic steatosis and cardiovascular disease (CVD). A randomized controlled trial including 43 NAFLD patients showed improvements in glycemic and lipid profiles, hepatic steatosis and fibrosis in those assigned to a low free sugars diet rather than a usual diet for 12 weeks [24]. After restricting free sugars intake to less than 3% of daily energy for 8 weeks, the hepatic fat fraction and alanine aminotransferase level were significantly decreased in 20 adolescent boys with biopsy-confirmed NAFLD [25]. In addition, in a cross-sectional study of 6613 participants, dietary fiber consumption was reported to be negatively associated with NAFLD as diagnosed by the fatty liver index [26]. In addition, replacement of free sugars with wholegrain starch and non-free sugars has been found to be protective for incident CVD in the UK Biobank cohort [10].

Two major points distinguish our study from the aforementioned studies. First, previous studies set hepatic steatosis as the outcome [24–26]. NAFLD is a benign condition for most patients [27]. Patients with a poor prognosis are of primary concern [28]. In this study, we focused on the long-term outcomes of NAFLD patients, including ESLD and mortality. Second, these studies explored the association of total and individual carbohydrate components with NAFLD. Without specific pharmacological therapies, diet control remains the first-line treatment for NAFLD, especially a calorie-restricted diet [8]. This study was the first to demonstrate that NAFLD patients can also benefit from substituting free sugars with non-free sugars, starch and fiber even when consuming the same amounts of calories. Of note, NAFLD patients with lower free sugars intake were found to have higher BMI or T2DM prevalence, which might mean those with risk factors for ESLD have switched their diet to a less free sugars pattern. However, those with higher free sugars intake still had a poorer prognosis, indicating that replacement free sugars with other carbohydrate components may represent an effective strategy to improve the long-term outcomes of NAFLD.

Although the biological mechanisms of different carbohydrate components and NAFLD have been poorly elucidated, several possible mechanisms have been proposed. The principal benefit of decreasing free sugars intake is a reduction in fructose intake from sugar-sweetened beverages [29]. Studies in humans and animals have reported that fructose promotes de novo lipogenesis and inhibits 𝛽-fatty acid oxidation in the liver [30–32]. Fructose metabolism in the intestine may destroy tight junctions and increase gut permeability, leading to the entry of endotoxin into the portal vein and triggering the formation of hepatic steatosis [33, 34]. In addition, endotoxemia contributes to the activation of the innate immune system [35]. In fructose-induced NAFLD models, fructose has been shown to modify the activity of NK cells and T cells [36]. Dietary fiber can improve insulin resistance and modulate hepatic lipid metabolism by producing short-chain fatty acids through intestinal microbial fermentation [37].

This study has several limitations. First, this was an observational study, and we could not exclude the effect of residual confounding, despite we conducted comprehensive adjustment for well-known risk factors. Second, dietary intake was only assessed at baseline, and we were unable to assess the dynamic change in dietary intake during follow-up. Nevertheless, this might not significantly change our results, since we observed a similar result when excluding those with an untypical diet. Third, NAFLD was defined by the fatty liver index in this study. Although the fatty liver index had a high sensitivity for the diagnosis of hepatic steatosis, abdominal imaging data would be preferable. Fourth, the use of self-reported dietary data will inevitably lead to measurement error and systematic bias due to underreporting [38], although the Oxford WebQ is a powerful tool for dietary assessment and the mean values of multiple 24-h dietary recall will represent habitual intake as much as possible [19]. Similarly, the information on alcohol consumption covered only 1 year prior to participation, which may not adequately reflect the drinking habit and is susceptible to reporting and recall bias. Fifth, participants enrolled in the current analysis were relatively healthy and affluent; hence, the number of outcomes will be underestimated, which may limit the generalizability of the results.

## Conclusions

In conclusion, this large prospective analysis showed that a higher intake of free sugars and a lower intake of non-free sugars or fiber were significantly associated with increased risks of ESLD and mortality of NAFLD. Furthermore, isocaloric replacement of free sugars with other carbohydrate components may confer health benefits of long-term outcomes of NAFLD. Our results suggest that NAFLD patients may benefit from changing the composition of dietary carbohydrate intake even with the same energy intake. When a low-carbohydrate diet is strongly recommended for patients with NAFLD, the composition of carbohydrate consumption should not be overlooked.

### Supplementary Information

**Additional file 1: Supplementary Table S1.** Description of types of dietary carbohydrates. **Supplementary Table S2.** Criteria for the end stage liver disease. **Supplementary Table S3.** Assessment of socioeconomic covariates. **Supplementary Table S4.** Spearman correlations between total carbohydrate and individual components. **Supplementary Table S5.** Characteristics of participants with NAFLD included in or excluded from the current study. **Supplementary Table S6.** Multivariable-adjusted HRs (95% CIs) of incident ESLD with non-free sugar from subgroup analyses. **Supplementary Table S7.** Multivariable-adjusted HRs (95% CIs) of incident ESLD with free sugar from subgroup analyses. **Supplementary Table S8.** Multivariable-adjusted HRs (95% CIs) of incident ESLD with dietary fiber from subgroup analyses. **Supplementary Table S9.** Multivariable-adjusted HRs (95% CIs) of all-cause mortality with non-free sugar from subgroup analyses. **Supplementary Table S10.** Multivariable-adjusted HRs (95% CIs) of all-cause mortality with free sugar from subgroup analyses. **Supplementary Table S11.** Multivariable-adjusted HRs (95% CIs) of all-cause mortality with dietary fiber from subgroup analyses. **Supplementary Table S12.** Sensitivity analyses of the HRs for the associations of different components of carbohydrate consumption with ESLD. **Supplementary Table S13.** Sensitivity analyses of the HRs for the associations of different components of carbohydrate consumption with all-cause mortality.

## Data Availability

The datasets analyzed during the current study are available in the UK Biobank Repository. This research has been conducted using the UK Biobank resource under application number 79302.

## Abbreviation

BMI: Body mass index
CVD: Cardiovascular disease
CI: Confidence interval
ESLD: End-stage liver disease
FLI: Fatty liver index
HR: Hazard ratio
ICD: International classification of diseases
NAFLD: Non-alcoholic fatty liver disease
