# Supplementary material for: Different dietary carbohydrate component intakes and long-term outcomes in patients with NAFLD: results of longitudinal analysis from the UK Biobank
Source: Nutr J. 2023 Dec 8;22:67. doi: 10.1186/s12937-023-00897-y (PMC10704713; doi:10.1186/s12937-023-00897-y)
Supplement: Supplementary file 1 — Additional file 1: Supplementary Table S1. Description of types of dietary carbohydrates. Supplementary Table S2. Criteria for the end stage liver disease. Supplementary Table S3. Assessment of socioeconomic covariates. Supplementary Table S4. Spearman correlations between total carbohydrate and individual components. Supplementary Table S5. Characteristics of participants with NAFLD included in or excluded from the current study. Supplementary Table S6. Multivariable-adjusted HRs (95% CIs) of incident ESLD with non-free sugar from subgroup analyses. Supplementary Table S7. Multivariable-adjusted HRs (95% CIs) of incident ESLD with free sugar from subgroup analyses. Supplementary Table S8. Multivariable-adjusted HRs (95% CIs) of incident ESLD with dietary fiber from subgroup analyses. Supplementary Table S9. Multivariable-adjusted HRs (95% CIs) of all-cause mortality with non-free sugar from subgroup analyses. Supplementary Table S10. Multivariable-adjusted HRs (95% CIs) of all-cause mortality with free sugar from subgroup analyses. Supplementary Table S11. Multivariable-adjusted HRs (95% CIs) of all-cause mortality with dietary fiber from subgroup analyses. Supplementary Table S12. Sensitivity analyses of the HRs for the associations of different components of carbohydrate consumption with ESLD. Supplementary Table S13. Sensitivity analyses of the HRs for the associations of different components of carbohydrate consumption with all-cause mortality. [file 12937_2023_897_MOESM1_ESM.docx]

**Supplementary Table S1. Description of types of dietary carbohydrates**

| **Carbohydrate type** | **Description** |
| --- | --- |
| Total carbohydrates | Carbohydrates from all foods and beverages. |
| Total sugars | Sugars from all foods and beverages. |
| Free sugars | All monosaccharides and disaccharides added to foods by the manufacturer, cook or consumer, plus sugars naturally present in honey, syrups and unsweetened fruit juices. Lactose when naturally present in milk and milk products is excluded. |
| Non-free sugars | Sugars naturally occurring in milk and dairy products, fresh and most types of processed fruit and vegetables and potatoes except for juices and purees, and cereal grains. Calculated as total sugars minus free sugars. |
| Starch | Starch from all foods. |
| Fibre | Non-starch polysaccharide, comprising cellulose and non-cellulose polysaccharides (e.g. pectins, glucans, arabinogalactans, arabinoxylans, gums and mucilages) by Englyst from all foods and beverages. |
|  | |

Kelly, R.K., Tong, T.Y.N., Watling, C.Z. *et al.* Associations between types and sources of dietary carbohydrates and cardiovascular disease risk: a prospective cohort study of UK Biobank participants. *BMC Med* **21**, 34 (2023). https://doi.org/10.1186/s12916-022-02712-7

**Supplementary Table S2. Criteria for the end stage liver disease**

| **Code** | **Description** |
| --- | --- |
| K74.6 | Other and unspecified cirrhosis of liver |
| K76.6 | Portal hypertension |
| K76.7 | Hepatorenal syndrome |
| I85.0 | Oesophageal varices with bleeding |
| I85.9 | Oesophageal varices without bleeding |
| I86.4 | Gastric varices |
| I98.2 | Oesophageal varices in diseases classified elsewhere |
| I98.3 | Oesophageal varices with bleeding in diseases classified elsewhere |
| R18 | Ascites |
| Z94.4 | Liver transplant |
| C22.0 | Liver cell carcinoma |

**Supplementary Table S3. Assessment of socioeconomic covariates**

| **Covariates** | **Description** | **UKB data field** |
| --- | --- | --- |
| Age at recruitment | This is a derived variable based on date of birth and date of attending an initial assessment center, truncated to whole year. | 21022 |
| Sex | Self-reported | 31 |
| Ethnicity | Participants were asked the questions “What is your ethnicity?” and “What is your ethnic background?” and answers were used to classify participants as white (British, Irish, Any other white background) and others (mixed background, Asian or Asian British, black or black British, other ethnic group, or unknown). | 21000 |
| Education level | Participant were asked “which of the following qualifications do you have?” at recruitment and the choices are as follows: College or University degree, A levels/AS levels or equivalent, O levels/GCSEs or equivalent, CSEs or equivalent, NVQ or HND or HNC or equivalent, Other professional qualifications eg: nursing, teaching, none of the above, and prefer not to answer.  We divided them into College/University degree or others. | 6138 |
| Household income | Participant were asked “What is the average total income before tax received by your HOUSEHOLD?" and the choices are as follows: less than 18,000, 18,000 to 30,999, 31,000 to 51,999, 52,000 to 100,000, greater than 100,000, do not know, prefer not to answer. | 738 |
| Smoking status | Self-reported smoking status was used to classify participants as never smokers, former smokers, or current smokers. | 20116 |
| Townsend deprivation index | Townsend deprivation index calculated immediately prior to participant joining UK Biobank. Based on the preceding national census output areas. Each participant is assigned a score corresponding to the output area in which their postcode is located. | 189 |

**Supplementary Table S4. Spearman correlations between total carbohydrate and individual components**

|  | Carbohydrate | Non-free sugar | Free sugar | Starch | Fiber |
| --- | --- | --- | --- | --- | --- |
| Carbohydrate | 1.00 | 0.43 | 0.33 | 0.41 | 0.23 |
| Non-free sugar |  | 1.00 | -0.24 | -0.16 | 0.35 |
| Free-sugar |  |  | 1.00 | -0.24 | -0.14 |
| Starch |  |  |  | 1.00 | 0.11 |
| Fiber |  |  |  |  | 1.00 |

All *P<*0.0**1**

**Supplementary Table S5. Characteristics of participants with NAFLD included in or excluded from the current study**

| Variables | Included  (*n*=26729) | Excluded  (*n*=21784) | *P* value |
| --- | --- | --- | --- |
| Male (%) | 62.0 | 61.0 | 0.043 |
| Age (years) | 57.0±7.6 | 56.8±7.9 | 0.002 |
| White ethnicity (%) | 96.0 | 92.7 | <0.001 |
| Townsend deprivation index | -1.5±2.9 | -1.2±3.1 | <0.001 |
| College or university degree (%) | 38.8 | 30.3 | <0.001 |
| Household income (£) |  |  | <0.001 |
| <18,000 | 16.5 | 20.0 |  |
| 18,000 to 30,999 | 23.4 | 23.3 |  |
| 31,000 to 51,999 | 26.3 | 23.4 |  |
| 52,000 to 100,000 | 20.0 | 17.2 |  |
| >100,000 | 4.6 | 3.5 |  |
| Sedentary behavior (%) | 44.2 | 42.6 | 0.628 |
| Smoking status (%) |  |  | <0.001 |
| Never | 54.7 | 52.8 |  |
| Previous | 38.5 | 37.4 |  |
| Current | 6.8 | 9.1 |  |
| Alcohol consumption (%) |  |  | <0.001 |
| Never or special occasions only | 22.6 | 26.6 |  |
| 1 to 3 times/month | 15.9 | 15.9 |  |
| 1 to 4 times/week | 52.8 | 50.3 |  |
| Daily or almost daily | 8.7 | 7.2 |  |
| Body mass index (kg/m^2^) | 31.4±4.5 | 31.7±4.6 | 0.001 |
| Waist circumference (cm) | 102.3±9.9 | 102.6±10.0 | 0.003 |
| Hypertension (%) | 68.3 | 67.5 | 0.053 |
| Diabetes (%) | 10.1 | 11.7 | <0.001 |
| Alanine aminotransferase (U/L) | 29.4±15.9 | 29.6±17.8 | <0.001 |
| Gamma glutamyltransferase (U/L) | 47.6±43.8 | 49.6±48.2 | <0.001 |
| Triglycerides (mmol/L) | 2.4±1.2 | 2.4±1.2 | <0.001 |
| Total cholesterol (mmol/L) | 5.7±1.2 | 5.7±1.2 | 0.167 |
| Total energy intake (kcal) | 2088.8±541.2 | 2088.1±764.0 | 0.908 |

**Supplementary Table S6. Multivariable-adjusted HRs (95% CIs) of incident ESLD with non-free sugar from subgroup analyses**

| Subgroups | Q1 | Q2 | Q3 | Q4 |  | *P*_interaction_ |
| --- | --- | --- | --- | --- | --- | --- |
| Gender |  |  |  |  |  | 0.391 |
| Male | 1 (ref) | 0.69 (0.46–1.04) | 0.75 (0.50–1.13) | 0.55 (0.33–0.90) |  |  |
| Female | 1 (ref) | 0.69 (0.40–1.18) | 0.63 (0.37–1.09) | 0.49 (0.27–0.88) |  |  |
| Age |  |  |  |  |  | 0.714 |
| ≤60 years | 1 (ref) | 0.44 (0.26–0.77) | 0.64 (0.39–1.05) | 0.67 (0.39–1.14) |  |  |
| >60 years | 1 (ref) | 0.94 (0.60–1.46) | 0.78 (0.49–1.24) | 0.45 (0.27–0.77) |  |  |
| Townsend index |  |  |  |  |  | 0.749 |
| Below median | 1 (ref) | 0.77 (0.49–1.21) | 0.87 (0.56–1.37) | 0.48 (0.29–0.82) |  |  |
| Above median | 1 (ref) | 0.63 (0.39–1.00) | 0.54 (0.33–0.88) | 0.56 (0.33–0.96) |  |  |
| Education level |  |  |  |  |  | 0.583 |
| Others | 1 (ref) | 0.63 (0.42–0.94) | 0.69 (0.46–1.03) | 0.53 (0.33–0.84) |  |  |
| College | 1 (ref) | 0.87 (0.49–1.55) | 0.79 (0.45–1.40) | 0.53 (0.28–1.00) |  |  |
| Smoking |  |  |  |  |  | 0.415 |
| Current or previous | 1 (ref) | 0.60 (0.38–0.94) | 0.74 (0.48–1.13) | 0.55 (0.34–0.91) |  |  |
| Never | 1 (ref) | 0.77 (0.48–1.24) | 0.66 (0.40–1.10) | 0.48 (0.27–0.85) |  |  |
| Obesity |  |  |  |  |  | 0.709 |
| No | 1 (ref) | 0.69 (0.38–1.25) | 0.52 (0.27–0.97) | 0.55 (0.26–1.15) |  |  |
| Yes | 1 (ref) | 0.70 (0.48–1.04) | 0.77 (0.52–1.13) | 0.50 (0.32–0.78) |  |  |
| Diabetes |  |  |  |  |  | 0.437 |
| No | 1 (ref) | 0.78 (0.54–1.11) | 0.68 (0.47–0.99) | 0.51 (0.33–0.78) |  |  |
| Yes | 1 (ref) | 0.44 (0.20–0.94) | 0.70 (0.35–1.38) | 0.51 (0.23–1.14) |  |  |

HRs were adjusted for age, sex, ethnicity, Townsend deprivation index, education level, household income, employment status, self-reported smoking status, sedentary behavior, body mass index, baseline diabetes, baseline hypertension, serum alanine aminotransferase, triglycerides, cholesterol levels, total energy intake, and total carbohydrate intake.

**Supplementary Table S7. Multivariable-adjusted HRs (95% CIs) of incident ESLD with free sugar from subgroup analyses**

| Subgroups | Q1 | Q2 | Q3 | Q4 |  | *P*_interaction_ |
| --- | --- | --- | --- | --- | --- | --- |
| Gender |  |  |  |  |  | 0.419 |
| Male | 1 (ref) | 0.96 (0.61–1.53) | 1.18 (0.75–1.84) | 1.29 (0.80–2.06) |  |  |
| Female | 1 (ref) | 1.81 (1.09–3.00) | 1.27 (0.70–2.31) | 2.25 (1.26–4.00) |  |  |
| Age |  |  |  |  |  | 0.662 |
| ≤60 years | 1 (ref) | 1.19 (0.69–2.05) | 1.34 (0.75–2.38) | 1.89 (1.07–3.34) |  |  |
| >60 years | 1 (ref) | 1.38 (0.89–2.14) | 1.23 (0.78–1.94) | 1.51 (0.93–2.47) |  |  |
| Townsend index |  |  |  |  |  | 0.901 |
| Below median | 1 (ref) | 1.26 (0.79–2.00) | 1.02 (0.63–1.65) | 1.47 (0.89–2.44) |  |  |
| Above median | 1 (ref) | 1.32 (0.79–2.19) | 1.58 (0.94–2.66) | 1.85 (1.09–3.16) |  |  |
| Education level |  |  |  |  |  | 0.631 |
| Others | 1 (ref) | 1.40 (0.92–2.13) | 1.21 (0.77–1.88) | 1.67 (1.06–2.62) |  |  |
| College | 1 (ref) | 1.17 (0.65–2.10) | 1.33 (0.73–2.42) | 1.55 (0.82–2.93) |  |  |
| Smoking |  |  |  |  |  | 0.567 |
| Current or previous | 1 (ref) | 1.79 (1.14–2.80) | 1.24 (0.76–2.02) | 1.54 (0.93–2.56) |  |  |
| Never | 1 (ref) | 0.92 (0.54–1.54) | 1.26 (0.75–2.12) | 1.76 (1.03–3.01) |  |  |
| Obesity |  |  |  |  |  | 0.423 |
| No | 1 (ref) | 1.84 (0.89–3.81) | 1.63 (0.77–3.45) | 1.81 (0.83–3.96) |  |  |
| Yes | 1 (ref) | 1.17 (0.79–1.73) | 1.17 (0.77–1.76) | 1.63 (1.07–2.50) |  |  |
| Diabetes |  |  |  |  |  | 0.660 |
| No | 1 (ref) | 1.31 (0.87–1.98) | 1.34 (0.89–2.03) | 1.64 (1.07–2.51) |  |  |
| Yes | 1 (ref) | 1.23 (0.65–2.32) | 0.93 (0.42–2.06) | 1.90 (0.91–3.96) |  |  |

HRs were adjusted for age, sex, ethnicity, Townsend deprivation index, education level, household income, employment status, self-reported smoking status, sedentary behavior, body mass index, baseline diabetes, baseline hypertension, serum alanine aminotransferase, triglycerides, cholesterol levels, total energy intake, and total carbohydrate intake.

**Supplementary Table S8. Multivariable-adjusted HRs (95% CIs) of incident ESLD with dietary fiber from subgroup analyses**

| Subgroups | Q1 | Q2 | Q3 | Q4 |  | *P*_interaction_ |
| --- | --- | --- | --- | --- | --- | --- |
| Gender |  |  |  |  |  | 0.571 |
| Male | 1 (ref) | 0.82 (0.53–1.25) | 0.75 (0.48–1.18) | 0.59 (0.35–1.00) |  |  |
| Female | 1 (ref) | 0.85 (0.53–1.37) | 0.58 (0.34–0.98) | 0.51 (0.26–0.99) |  |  |
| Age |  |  |  |  |  | 0.617 |
| ≤60 years | 1 (ref) | 0.74 (0.46–1.18) | 0.60 (0.35–1.02) | 0.40 (0.21–0.76) |  |  |
| >60 years | 1 (ref) | 0.90 (0.59–1.39) | 0.75 (0.48–1.19) | 0.70 (0.40–1.22) |  |  |
| Townsend index |  |  |  |  |  | 0.715 |
| Below median | 1 (ref) | 0.94 (0.60–1.48) | 0.74 (0.46–1.20) | 0.62 (0.35–1.10) |  |  |
| Above median | 1 (ref) | 0.71 (0.45–1.12) | 0.63 (0.38–1.04) | 0.51 (0.28–0.94) |  |  |
| Education level |  |  |  |  |  | 0.205 |
| Others | 1 (ref) | 0.74 (0.50–1.10) | 0.73 (0.48–1.12) | 0.56 (0.34–0.93) |  |  |
| College | 1 (ref) | 0.95 (0.56–1.62) | 0.58 (0.32–1.04) | 0.53 (0.25–1.12) |  |  |
| Smoking |  |  |  |  |  | 0.525 |
| Current or previous | 1 (ref) | 0.78 (0.49–1.24) | 0.82 (0.51–1.31) | 0.63 (0.35–1.13) |  |  |
| Never | 1 (ref) | 0.84 (0.54–1.31) | 0.56 (0.34–0.93) | 0.48 (0.26–0.88) |  |  |
| Obesity |  |  |  |  |  | 0.246 |
| No | 1 (ref) | 0.79 (0.41–1.52) | 0.71 (0.35–1.43) | 0.83 (0.38–1.83) |  |  |
| Yes | 1 (ref) | 0.84 (0.58–1.20) | 0.66 (0.45–0.99) | 0.46 (0.28–0.76) |  |  |
| Diabetes |  |  |  |  |  | 0.969 |
| No | 1 (ref) | 0.88 (0.61–1.26) | 0.69 (0.46–1.03) | 0.62 (0.38–1.00) |  |  |
| Yes | 1 (ref) | 0.63 (0.33–1.21) | 0.57 (0.29–1.14) | 0.36 (0.15–0.83) |  |  |

HRs were adjusted for age, sex, ethnicity, Townsend deprivation index, education level, household income, employment status, self-reported smoking status, sedentary behavior, body mass index, baseline diabetes, baseline hypertension, serum alanine aminotransferase, triglycerides, cholesterol levels, total energy intake, and total carbohydrate intake.

**Supplementary Table S9. Multivariable-adjusted HRs (95% CIs) of all-cause mortality with non-free sugar from subgroup analyses**

| Subgroups | Q1 | Q2 | Q3 | Q4 |  | *P*_interaction_ |
| --- | --- | --- | --- | --- | --- | --- |
| Gender |  |  |  |  |  | 0.751 |
| Male | 1 (ref) | 0.92 (0.79–1.07) | 0.85 (0.72–1.00) | 0.81 (0.67–0.97) |  |  |
| Female | 1 (ref) | 0.96 (0.73–1.27) | 0.85 (0.64–1.12) | 0.78 (0.59–1.04) |  |  |
| Age |  |  |  |  |  | 0.875 |
| ≤60 years | 1 (ref) | 1.04 (0.82–1.31) | 1.04 (0.82–1.33) | 0.86 (0.65–1.14) |  |  |
| >60 years | 1 (ref) | 0.88 (0.75–1.04) | 0.77 (0.65–0.92) | 0.77 (0.64–0.92) |  |  |
| Townsend index |  |  |  |  |  | 0.773 |
| Below median | 1 (ref) | 0.96 (0.79–1.17) | 0.87 (0.71–1.07) | 0.84 (0.67–1.05) |  |  |
| Above median | 1 (ref) | 0.91 (0.75–1.10) | 0.83 (0.68–1.01) | 0.77 (0.62–0.96) |  |  |
| Education level |  | . |  |  |  | 0.948 |
| Others | 1 (ref) | 0.97 (0.83–1.15) | 0.83 (0.70–0.99) | 0.83 (0.69–1.00) |  |  |
| College | 1 (ref) | 0.85 (0.67–1.08) | 0.88 (0.69–1.12) | 0.75 (0.57–0.98) |  |  |
| Smoking |  |  |  |  |  | 0.815 |
| Current or previous | 1 (ref) | 0.88 (0.74–1.04) | 0.85 (0.71–1.01) | 0.80 (0.66–0.98) |  |  |
| Never | 1 (ref) | 1.02 (0.82–1.27) | 0.85 (0.68–1.07) | 0.79 (0.62–1.02) |  |  |
| Obesity |  |  |  |  |  | 0.908 |
| No | 1 (ref) | 0.87 (0.68–1.12) | 0.92 (0.71–1.18) | 0.80 (0.60–1.07) |  |  |
| Yes | 1 (ref) | 0.96 (0.82–1.13) | 0.83 (0.70–0.98) | 0.80 (0.67–0.96) |  |  |
| Diabetes |  |  |  |  |  | 0.189 |
| No | 1 (ref) | 0.93 (0.80–1.08) | 0.86 (0.74–1.01) | 0.81 (0.68–0.96) |  |  |
| Yes | 1 (ref) | 0.94 (0.70–1.27) | 0.80 (0.59–1.09) | 0.74 (0.52–1.04) |  |  |

HRs were adjusted for age, sex, ethnicity, Townsend deprivation index, education level, household income, employment status, self-reported smoking status, sedentary behavior, body mass index, baseline diabetes, baseline hypertension, serum alanine aminotransferase, triglycerides, cholesterol levels, total energy intake, and total carbohydrate intake.

**Supplementary Table S10. Multivariable-adjusted HRs (95% CIs) of all-cause mortality with free sugar from subgroup analyses**

| Subgroups | Q1 | Q2 | Q3 | Q4 |  | *P*_interaction_ |
| --- | --- | --- | --- | --- | --- | --- |
| Gender |  |  |  |  |  | 0.866 |
| Male | 1 (ref) | 1.14 (0.97–1.35) | 1.09 (0.92–1.29) | 1.23 (1.03–1.47) |  |  |
| Female | 1 (ref) | 0.97 (0.77–1.23) | 1.10 (0.87–1.41) | 1.16 (0.89–1.50) |  |  |
| Age |  |  |  |  |  | 0.664 |
| ≤60 years | 1 (ref) | 0.98 (0.76–1.26) | 1.13 (0.88–1.44) | 1.13 (0.88–1.46) |  |  |
| >60 years | 1 (ref) | 1.13 (0.96–1.33) | 1.07 (0.90–1.27) | 1.23 (1.03–1.47) |  |  |
| Townsend index |  |  |  |  |  | 0.968 |
| Below median | 1 (ref) | 1.24 (1.02–1.50) | 1.06 (0.86–1.29) | 1.28 (1.04–1.59) |  |  |
| Above median | 1 (ref) | 0.94 (0.77–1.14) | 1.13 (0.93–1.37) | 1.14 (0.93–1.39) |  |  |
| Education level |  |  |  |  |  | 0.600 |
| Others | 1 (ref) | 1.11 (0.93–1.31) | 1.07 (0.90–1.27) | 1.18 (0.99–1.41) |  |  |
| College | 1 (ref) | 1.04 (0.83–1.30) | 1.11 (0.87–1.40) | 1.22 (0.95–1.57) |  |  |
| Smoking |  |  |  |  |  | 0.137 |
| Current or previous | 1 (ref) | 1.11 (0.93–1.32) | 1.11 (0.93–1.33) | 1.16 (0.96–1.40) |  |  |
| Never | 1 (ref) | 1.05 (0.84–1.31) | 1.04 (0.83–1.31) | 1.27 (1.01–1.59) |  |  |
| Obesity |  |  |  |  |  | 0.940 |
| No | 1 (ref) | 1.07 (0.82–1.40) | 1.05 (0.80–1.38) | 1.21 (0.92–1.58) |  |  |
| Yes | 1 (ref) | 1.09 (0.93–1.28) | 1.10 (0.93–1.30) | 1.20 (1.01–1.43) |  |  |
| Diabetes |  |  |  |  |  | 0.503 |
| No | 1 (ref) | 1.05 (0.90–1.24) | 1.12 (0.96–1.32) | 1.23 (1.04–1.45) |  |  |
| Yes | 1 (ref) | 1.21 (0.93–1.57) | 0.92 (0.67–1.27) | 1.14 (0.82–1.60) |  |  |

HRs were adjusted for age, sex, ethnicity, Townsend deprivation index, education level, household income, employment status, self-reported smoking status, sedentary behavior, body mass index, baseline diabetes, baseline hypertension, serum alanine aminotransferase, triglycerides, cholesterol levels, total energy intake, and total carbohydrate intake.

**Supplementary Table S11. Multivariable-adjusted HRs (95% CIs) of all-cause mortality with dietary fiber from subgroup analyses**

| Subgroups | Q1 | Q2 | Q3 | Q4 |  | *P*_interaction_ |
| --- | --- | --- | --- | --- | --- | --- |
| Gender |  |  |  |  |  | 0.819 |
| Male | 1 (ref) | 1.00 (0.84–1.19) | 0.92 (0.77–1.10) | 0.79 (0.64–0.97) |  |  |
| Female | 1 (ref) | 0.86 (0.68–1.10) | 0.80 (0.62–1.05) | 0.85 (0.63–1.15) |  |  |
| Age |  |  |  |  |  | 0.897 |
| ≤60 years | 1 (ref) | 0.89 (0.70–1.13) | 0.84 (0.65–1.09) | 0.82 (0.62–1.10) |  |  |
| >60 years | 1 (ref) | 0.99 (0.84–1.18) | 0.91 (0.76–1.09) | 0.81 (0.66–0.99) |  |  |
| Townsend index |  |  |  |  |  | 0.012 |
| Below median | 1 (ref) | 1.09 (0.89–1.33) | 1.00 (0.80–1.24) | 0.96 (0.76–1.23) |  |  |
| Above median | 1 (ref) | 0.86 (0.71–1.05) | 0.81 (0.66–0.99) | 0.69 (0.54–0.87) |  |  |
| Education level |  |  |  |  |  | 0.150 |
| Others | 1 (ref) | 0.94 (0.79–1.11) | 0.94 (0.78–1.12) | 0.83 (0.67–1.02) |  |  |
| College | 1 (ref) | 0.99 (0.78–1.25) | 0.78 (0.60–1.01) | 0.77 (0.57–1.02) |  |  |
| Smoking |  |  |  |  |  | 0.075 |
| Current or previous | 1 (ref) | 0.97 (0.81–1.15) | 0.89 (0.74–1.08) | 0.82 (0.66–1.02) |  |  |
| Never | 1 (ref) | 0.95 (0.76–1.19) | 0.87 (0.69–1.10) | 0.78 (0.60–1.02) |  |  |
| Obesity |  |  |  |  |  | 0.633 |
| No | 1 (ref) | 0.83 (0.64–1.08) | 0.78 (0.60–1.03) | 0.83 (0.61–1.12) |  |  |
| Yes | 1 (ref) | 1.01 (0.86–1.19) | 0.93 (0.78–1.11) | 0.80 (0.65–0.97) |  |  |
| Diabetes |  |  |  |  |  | 0.983 |
| No | 1 (ref) | 0.95 (0.81–1.10) | 0.88 (0.75–1.04) | 0.81 (0.67–0.97) |  |  |
| Yes | 1 (ref) | 0.99 (0.73–1.36) | 0.88 (0.64–1.23) | 0.77 (0.53–1.12) |  |  |

HRs were adjusted for age, sex, ethnicity, Townsend deprivation index, education level, household income, employment status, self-reported smoking status, sedentary behavior, body mass index, baseline diabetes, baseline hypertension, serum alanine aminotransferase, triglycerides, cholesterol levels, total energy intake, and total carbohydrate intake.

**Supplementary Table S12. Sensitivity analyses of the HRs for the associations of different components of carbohydrate consumption with ESLD**

|  | Exclude the first 2 years  of follow-up | Exclude extreme BMIs | Exclude diet not typical | Exclude extreme energy intake |  |
| --- | --- | --- | --- | --- | --- |
| Non-free sugar |  |  |  |  |  |
| Q1 | 1 (ref) | 1 (ref) | 1 (ref) | 1 (ref) |  |
| Q2 | 0.66 (0.47–0.91) | 0.63 (0.45–0.89) | 0.72 (0.52–0.99) | 0.66 (0.48–0.91) |  |
| Q3 | 0.65 (0.47–0.91) | 0.66 (0.47–0.92) | 0.61 (0.43–0.85) | 0.68 (0.50–0.94) |  |
| Q4 | 0.48 (0.33–0.69) | 0.48 (0.33–0.71) | 0.60 (0.42–0.85) | 0.52 (0.37–0.75) |  |
| Free sugar |  |  |  |  |  |
| Q1 | 1 (ref) | 1 (ref) | 1 (ref) | 1 (ref) |  |
| Q2 | 1.30 (0.93–1.81) | 1.28 (0.91–1.81) | 1.27 (0.91–1.79) | 1.36 (0.98–1.88) |  |
| Q3 | 1.18 (0.83–1.68) | 1.21 (0.85–1.73) | 1.47 (1.04–2.07) | 1.32 (0.94–1.86) |  |
| Q4 | 1.59 (1.12–2.26) | 1.52 (1.06–2.18) | 1.67 (1.17–2.37) | 1.60 (1.13–2.26) |  |
| Starch |  |  |  |  |  |
| Q1 | 1 (ref) | 1 (ref) | 1 (ref) | 1 (ref) |  |
| Q2 | 0.87 (0.62–1.21) | 0.87 (0.62–1.21) | 0.87 (0.62–1.21) | 0.87 (0.62–1.21) |  |
| Q3 | 0.96 (0.69–1.33) | 0.96 (0.69–1.33) | 0.96 (0.69–1.33) | 0.96 (0.69–1.33) |  |
| Q4 | 1.02 (0.73–1.43) | 1.02 (0.73–1.43) | 1.02 (0.73–1.43) | 1.02 (0.73–1.43) |  |
| Fiber |  |  |  |  |  |
| Q1 | 1 (ref) | 1 (ref) | 1 (ref) | 1 (ref) |  |
| Q2 | 0.85 (0.62–1.17) | 0.86 (0.62–1.20) | 0.81 (0.59–1.12) | 0.86 (0.63–1.18) |  |
| Q3 | 0.68 (0.48–0.97) | 0.71 (0.49–1.02) | 0.73 (0.52–1.03) | 0.66 (0.47–0.93) |  |
| Q4 | 0.57 (0.37–0.86) | 0.59 (0.39–0.91) | 0.57 (0.38–0.85) | 0.57 (0.38–0.85) |  |

HRs were adjusted for age, sex, ethnicity, Townsend deprivation index, education level, household income, employment status, self-reported smoking status, sedentary behavior, body mass index, baseline diabetes, baseline hypertension, serum alanine aminotransferase, triglycerides, cholesterol levels, total energy intake, and total carbohydrate intake.

**Supplementary Table S13. Sensitivity analyses of the HRs for the associations of different components of carbohydrate consumption with all-cause mortality**

|  | Exclude the first 2 years  of follow-up | Exclude extreme BMIs | Exclude diet not typical | Exclude extreme energy intake |  |
| --- | --- | --- | --- | --- | --- |
| Non-free sugar |  |  |  |  |  |
| Q1 | 1 (ref) | 1 (ref) | 1 (ref) | 1 (ref) |  |
| Q2 | 0.93 (0.81–1.06) | 0.91 (0.79–1.05) | 0.92 (0.80–1.05) | 0.94 (0.82–1.07) |  |
| Q3 | 0.85 (0.73–0.97) | 0.84 (0.73–0.97) | 0.87 (0.75–1.00) | 0.86 (0.75–0.99) |  |
| Q4 | 0.80 (0.68–0.93) | 0.79 (0.67–0.93) | 0.77 (0.66–0.90) | 0.81 (0.69–0.94) |  |
| Free sugar |  |  |  |  |  |
| Q1 | 1 (ref) | 1 (ref) | 1 (ref) | 1 (ref) |  |
| Q2 | 1.08 (0.94–1.24) | 1.09 (0.95–1.26) | 1.10 (0.96–1.26) | 1.09 (0.95–1.25) |  |
| Q3 | 1.07 (0.93–1.23) | 1.05 (0.90–1.21) | 1.05 (0.91–1.21) | 1.07 (0.93–1.23) |  |
| Q4 | 1.20 (1.04–1.39) | 1.20 (1.03–1.39) | 1.17 (1.01–1.35) | 1.20 (1.03–1.38) |  |
| Starch |  |  |  |  |  |
| Q1 | 1 (ref) | 1 (ref) | 1 (ref) | 1 (ref) |  |
| Q2 | 0.87 (0.75–0.99) | 0.87 (0.75–0.99) | 0.87 (0.75–0.99) | 0.87 (0.75–0.99) |  |
| Q3 | 0.98 (0.86–1.13) | 0.98 (0.86–1.13) | 0.98 (0.86–1.13) | 0.98 (0.86–1.13) |  |
| Q4 | 0.94 (0.81–1.08) | 0.94 (0.81–1.08) | 0.94 (0.81–1.08) | 0.94 (0.81–1.08) |  |
| Fiber |  |  |  |  |  |
| Q1 | 1 (ref) | 1 (ref) | 1 (ref) | 1 (ref) |  |
| Q2 | 0.97 (0.84–1.11) | 0.90 (0.78–1.04) | 0.91 (0.79–1.05) | 0.91 (0.79–1.05) |  |
| Q3 | 0.88 (0.76–1.02) | 0.85 (0.73–0.99) | 0.81 (0.70–0.95) | 0.85 (0.73–0.98) |  |
| Q4 | 0.81 (0.69–0.96) | 0.78 (0.66–0.93) | 0.83 (0.70–0.98) | 0.79 (0.67–0.93) |  |

HRs were adjusted for age, sex, ethnicity, Townsend deprivation index, education level, household income, employment status, self-reported smoking status, sedentary behavior, body mass index, baseline diabetes, baseline hypertension, serum alanine aminotransferase, triglycerides, cholesterol levels, total energy intake, and total carbohydrate intake.
